# Noise-Aware and Light-Weight VLSI Design of Bilateral Filter for Robust and Fast Image Denoising in Mobile Systems

**DOI:** 10.3390/s20174722

**Published:** 2020-08-21

**Authors:** Sung-Joon Jang, Youngbae Hwang

**Affiliations:** 1 Intelligent Image Processing Research Center, Korea Electronics Technology Institute, Seongnam 13509, Korea; sjjang0626@keti.re.kr; 2 Department of Electronics Engineering, Chungbuk National University, Cheongju 28644, Korea

**Keywords:** image denoising, bilateral filter, range kernel, image noise model, very-large-scale integration (VLSI), field-programmable gate array (FPGA)

## Abstract

The range kernel of bilateral filter degrades image quality unintentionally in real environments because the pixel intensity varies randomly due to the noise that is generated in image sensors. Furthermore, the range kernel increases the complexity due to the comparisons with neighboring pixels and the multiplications with the corresponding weights. In this paper, we propose a noise-aware range kernel, which estimates noise using an intensity difference-based image noise model and dynamically adjusts weights according to the estimated noise, in order to alleviate the quality degradation of bilateral filters by noise. In addition, to significantly reduce the complexity, an approximation scheme is introduced, which converts the proposed noise-aware range kernel into a binary kernel while using the statistical hypothesis test method. Finally, blue a fully parallelized and pipelined very-large-scale integration (VLSI) architecture of a noise-aware bilateral filter (NABF) that is based on the proposed binary range kernel is presented, which was successfully implemented in field-programmable gate array (FPGA). The experimental results show that the proposed NABF is more robust to noise than the conventional bilateral filter under various noise conditions. Furthermore, the proposed VLSI design of the NABF achieves 10.5 and 95.7 times higher throughput and uses 63.6–97.5% less internal memory than state-of-the-art bilateral filter designs.

## 1. Introduction

Image denoising methods have been rapidly evolved as a critical component of image processing pipeline [1] and high-level vision tasks [2,3]. Recently introduced methods, such as improved block-matching and three-dimensional (3D) filtering (BM3D) [4] and deep learning-based algorithms [5,6], reduce a noise significantly under various environments. However, these methods are infeasible for battery-powered mobile systems, in which the low-power operation is essential, owing to high complexity requiring high-end central processing units (CPUs) or graphics processing units (GPUs). Alternatively, the bilateral filter [7] has been started to adopt for mobile systems [8,9], owing to relatively low complexity and edge-preserving characteristic. Furthermore, very-large-scale integration (VLSI) designs for real-time and low-power bilateral filtering in mobile systems have been recently presented [10,11,12,13].

However, the bilateral filter is problematic to use practically owing to image quality degradation by noise. This is because the pixel intensity is changed randomly by the noise that is generated in image sensors [14], but the range kernel cannot distinguish whether this variation is caused by noise or not. Furthermore, a non-optimal parameter defined by a user can degrade the quality of filtered image.

In addition, VLSI designs of the bilateral filter suffer from increased complexity due to the range kernel. The range kernel is typically implemented using a look-up table (LUT) memory, which stores pre-defined weights according to the difference in pixel intensity. Because differences in pixel intensity are random, separate LUT memories are required for the parallel processing of pixels within a pixel window, and this increases the resource use in proportion to the pixel window size.

In this paper, we propose a noise-aware bilateral filter (NABF) to resolve the aforementioned image quality degradation problem, which replaces the conventional range kernel with an intensity difference-based image noise model [15] to estimate the probability that an intensity difference is caused by noise and adjusts the weight dynamically according to the estimated noise probability. A user-defined parameter that affects the quality of the bilateral filter is also eliminated by this replacement. In addition, the proposed range kernel is approximated into a binary kernel using the statistical hypothesis test method to reduce the usage of the LUT memory and the logic considerably in a VLSI design [16]. Finally, a fully parallelized and pipelined VLSI of the NABF based on the proposed binary range kernel is designed, which was successfully implemented in a field-programmable gate array (FPGA). The experimental results demonstrate that the proposed NABF and its approximated one are more robust to noise than the conventional bilateral filter under various noise conditions. Furthermore, the proposed VLSI design achieves 10.5 and 95.7 times higher throughput and uses 63.6–97.5% less internal memory when compared with recent VLSI designs of the bilateral filter.

The rest of this paper is organized, as follows. Related studies are presented in Section 2. Section 3 presents the NABF and its approximation method. Section 4 presents the VLSI architecture of the NABF. Experimental results are shown in Section 5. Lastly, Section 6 concludes the paper.

## 2. Related Works

### 2.1. Optimal Parameter Selection of Bilateral Filter

Because ground truth images do not exist in the real environment, statistical approaches, such as Stein’s unbiased risk estimator (SURE), Poisson unbiased risk estimator (PURE), and Chi-Square unbiased risk estimator (CURE), are adopted for optimal parameter selection of the bilateral filter [17,18,19]. SURE, PURE, and CURE use Gaussian noise, Poisson, and Chi-Square noise distribution, respectively. Furthermore, the output of bilateral filter is adopted as an estimate of the noiseless ground truth image, and unbiased estimated risk is modeled. Finally, the values of the parameters are selected by minimization of the estimated risk. Despite this, usefulness cannot be adopted for real-time mobile systems owing to high complexity. For example, the CURE requires dozens of minutes of execution time in a PC [20]. Recently, a training method that is based on neural network is presented [21]. Image texture features from training images and their optimal parameter are used for inputs of training model, and peak signal-to-noise ratio (PSNR) is adopted as a loss function. Furthermore, the two-dimensional (2D) wavelet transform and gray-level co-occurrence matrix are adopted to extract image texture features. Finally, a forward path of trained neural network is used as predictive model of optimal parameter for bilateral filtering. However, this method also requires a high-end CPU or GPU, owing to high complexity caused by neural network and feature extraction.

### 2.2. VLSI Design of Bilateral Filter

A VLSI design based on the integral histogram-based bilateral filter [22] is presented in order to process a large size of window in real-time [10]. However, their design requires many internal memories to store the histogram. Moreover, external memory access, which causes large power consumption and delay in mobile systems, is needed. In [11,12], the equation of bilateral filter is implemented to parallelized VLSI designs without modification. In particular, Gabiger-Rose et al. introduced a resource-efficient approach that divides pixels within the window into multiple groups and assigns each group to the separate pipeline [12]. Although this approach reduces the usage of resources, the processing speed is degraded and the large lookup memory to store weights is required. In [13], a VLSI that is based on a fast bilateral filtering method using an approximation [23] is designed to support arbitrary size of the window without the increase of resources. However, the speed is low owing to its iterative nature.

## 3. Proposed Approach

In order o preserve the details of images, the range kernel ($f_{r}$) of a bilateral filter controls the degree of smoothing by the spatial kernel ($f_{s}$) using the intensity difference between two pixels:

(1) $$\begin{array}{c} I_{BF}\left(x\right)\;\;=\;\;\frac{1}{W_{p}}\;\;\sum_{x_{i}\in Ω}\;I\left(x_{i}\right)\;\cdot \;f_{s}\;\cdot \;f_{r}\;,\\ where\;\;\;\;\;f_{s}=exp\left(-\frac{|x_{i}{-x|}^{2}}{2\sigma_{s}^{2}}\right),\;\;\;\;\;f_{r}=exp\left(-\frac{|I\left(x_{i}\right)-I\left(x\right){|}^{2}}{2\sigma_{r}^{2}}\right). \end{array}$$

Here, I(x) and $I_{BF}\left(x\right)$ are the pixel intensity in position *x*, and its filtered intensity, respectively, and Ω is a pixel window. In addition, $x_{i}\in Ω$ are neighboring pixels of *x* within a pixel window; $f_{s}$ and $f_{r}$ are the spatial and range kernel, respectively; $\sigma_{s}$ and $\sigma_{r}$ are standard deviations of $f_{s}$ and $f_{r}$, respectively, and $W_{p}$ is a normalization term. However, I(x) and $I(x_{i})$ can be changed randomly by the noise generated in image sensors [14], e.g., shot noise, but the range kernel ($f_{r}$) cannot recognize whether this change is caused by noise or not. A noise-aware bilateral filter is proposed to resolve this issue.

### 3.1. Noise-Aware Bilateral Filter (NABF)

An image noise model is adapted for estimating the noise at each pixel. In particular, the image noise model [15] that is based on the intensity difference between two pixels is adopted among various image noise models to determine whether the intensity difference, which is the input of the range kernel, is caused by noise or not:

(2) $$p\left(k;\mu \right)=exp^{-2\mu }\cdot B_{k}\cdot 2\mu .$$

Here, *k* is the intensity difference between two pixels; p(k) and μ are the noise probability function and its parameter, respectively; and, $B_{k}\left(\cdot \right)$ is the modified Bessel function of the first kind as:

(3) $$B_{k}\left(x\right)=\sum_{m=0}^{\infty }\frac{{\left(-1\right)}^{m}}{m!\Gamma (M+k+1)}{\left(\frac{x}{2}\right)}^{2m+k}.$$

**Figure 1:**
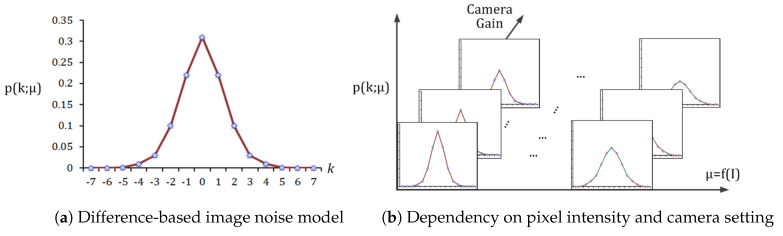
Intensity difference-based image noise model [15]. Here, *I* and *k* denote a pixel intensity and the difference in intensity between two random pixels, respectively; and, p(k) and μ are the noise probability function and its parameter, respectively.

The probability that an intensity difference is caused by noise is modeled, as shown in Figure 1a. This noise probability function is dependent on μ, as shown in Figure 1b. Furthermore, μ varies proportionally with the pixel intensity:

(4) $$\begin{array}{c} \mu =f\left(I\right)=c_{0}\cdot I+c_{1}, \end{array}$$

where *I* is a pixel intensity, and $c_{0}$ and $c_{1}$ are constants due to a camera setting such as a camera gain. By capturing the colorchecker board, there is the linear relationship between intensity and noise parameters for each homogeneous color patch [15]. From the line, the values of $c_{0}$ and $c_{1}$ can be determined by the fitting process. For details that are related to equations and their derivation, refer to the literatures [15].

The range kernel of (1) with the above difference-based image noise model is replaced, as expressed in (5), and the degree of smoothing by the spatial kernel is controlled according to the noise probability of intensity difference:

(5) $$\begin{array}{c} f_{r}^{NABF}=exp^{-2\mu }\cdot B_{|I\left(x_{i}\right)-I\left(x\right)|}\cdot 2\mu ,\\ where\;\;\;\;\;\mu =c_{0}\cdot I\left(x\right)+c_{1}. \end{array}$$

Here, $f_{r}^{NABF}$ is the noise-aware range kernel, and I(x) and $I(x_{i})$ are the intensities of a target pixel and a neighboring pixel, respectively. When the noise probability of intensity difference is small, the proposed range kernel judges that the difference is caused by a real scene change, and the neighboring pixel is excluded from the smoothing by assigning a smaller weight.

All of the noise probabilities according to $|I\left(x_{i}\right)-I\left(x\right)|$ and μ are obtained by calibration [15] and they are stored in LUTs in advance. Because the proposed range kernel refers to the noise probability simply from the LUTs using $|I\left(x_{i}\right)-I\left(x\right)|$ during operation without extra computations, the speed is not degraded in comparison with the conventional bilateral filter.

### 3.2. Binary Noise-Aware Bilateral Filter (B-NABF)

The quality degradation due to noise can be mitigated substantially by the proposed NABF. However, the NABF increases the LUT usage considerably owing to storing noise probability values corresponding to all intensity differences, μs, and camera gains. A binary NABF (B-NABF) which approximates (5) using the statistical hypothesis test is introduced to significantly reduce the computational complexity of the bilateral filter as well as the LUT usage[16]:

(6) $$\begin{array}{c} f_{r}^{B-NABF}\;=\;\left\{\begin{array}{cc} 1 & \mathrm{if}∥I\left(x_{i}\right)-I\left(x\right)∥\leq K_{C}\left(x\right),\\ 0 & \mathrm{otherwise}, \end{array}\right.\\ where\;\;\;\;\;K_{C}\left(x\right)=\arg\max A\left(k\right)\;\;s.t.\;A\left(k\right)\leq 1-\alpha ,\;\;\;\;\;A\left(k\right)=F\left(k;\mu \right)-F\left(-k;\mu \right). \end{array}$$

Here, $f_{r}^{B-NABF}$ is the binary noise-aware range kernel. I(x) and $I(x_{i})$ are the intensity of a target pixel and that of a neighboring pixel, respectively; $K_{C}\left(x\right)$ is the critical value corresponding to the I(x); *F* is the cumulative distribution function of the $f_{r}^{NABF}$; and α is the significance level, which is commonly set from 0.01 to 0.1 [16].

**Figure 2:**
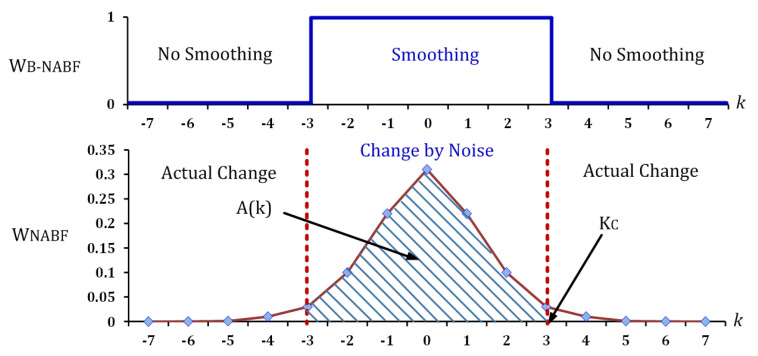
Binary noise-aware range kernel. Here, A[k] is the cumulative value of probability between two symmetric *k*s; $K_{C}$ denotes the critical value for a binary range kernel; and, $W_{NABF}$ and $W_{B-NABF}$ are weights by a noise-aware range kernel and a binary noise-aware range kernel, respectively.

The $K_{C}\left(x\right)$ value is determined to be a *k*, which is the cumulative value of $f_{r}^{NABF}$ between two symmetric *k*s (A[k]) that is closest to 1−α. A weight is assigned to 1 or 0 by comparing the intensity difference with $K_{C}\left(x\right)$, as shown in Figure 2. Consequently, the use of LUT is reduced because the B-NABF only requires $K_{C}$s instead of noise probability values at all intensity differences. Furthermore, the computational complexity is reduced owing to a binarization of weights.

To further reduce the LUT usage, only the $K_{C}$s of partial camera gains are stored in the LUTs instead of storing the $K_{C}$ values corresponding to all camera gains, and an interpolated $K_{C}$ for a new camera gain is generated, as follows:

(7) $$\begin{array}{c} K_{C}^{i}\;=\;R\;\left(\;\frac{K_{C}^{i↓}\times \left(G_{i↑}-G_{i}\right)\;+\;K_{C}^{i↑}\times \left(G_{i}-G_{i↓}\right)}{G_{i↑}\;-\;G_{i↓}}\;\right), \end{array}$$

where R(·) is a round operation;$K_{C}^{i}$ and $G_{i}$ are the critical value and camera gain of an index *i*, respectively; and, $G_{i↓}$ and $G_{i↑}$ are the closest lower and upper gains with *i*, respectively.

## 4. VLSI Design

**Figure 3:**
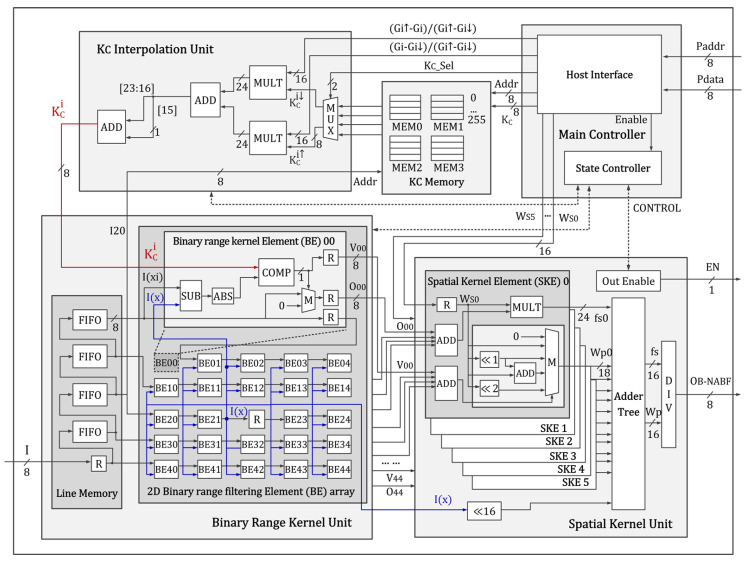
Proposed VLSI architecture of noise-aware bilateral filter. Here, R denotes registers; ADD and SUB denote adders and subtractors, respectively; MULT and DIV are multipliers and the divider, respectively; M and MUX are multiplexers; and ABS and COMP are the logic to conduct absolute operation and comparison, respectively.

A VLSI is designed to accelerate the proposed binary noise-aware bilateral filter. To process a 5×5-sized pixel window per clock, the hardware design is fully parallelized and pipelined. It mainly consists of the main controller, a binary range kernel unit to compute (6), a spatial kernel unit to perform $f_{s}$ of (1), a $K_{C}$ memory to store critical values, and a $K_{C}$ interpolation unit to calculate (7), as shown in Figure 3. The operation of each unit is described in detail, as follows.

### 4.1. Main Controller

**Figure 4:**
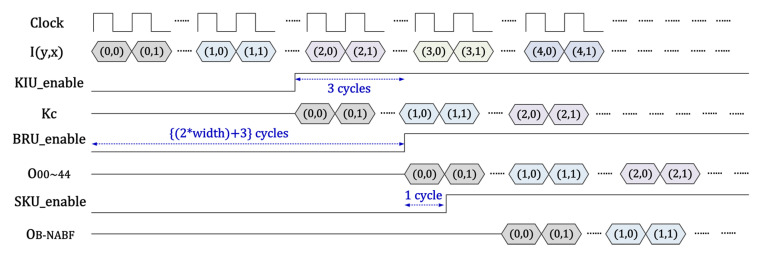
Control and timing diagram for pipelined operation. Here, I denotes the pixel intensity of an input image; x and y denote horizontal and vertical position in an image, respectively; KIU, BRU, and SKU are $K_{C}$ interpolation unit, binary range kernel unit, and spatial kernel unit, respectively; $K_{C}$, $O_{00}$∼$O_{44}$, and $O_{B-NABF}$ are outputs of KIU, BRU, and SKU, respectively.

The main controller comprises a state controller for handling the operational sequence of all units which are pipelined pixel by pixel, as shown in Figure 4, and a host interface module to communicate with the host central processing unit (CPU). First, the state controller activates the line memory in the binary range kernel unit for the line buffering of an image. The pixel intensity (*I*) of each line is sent to the two dimensional (2D) binary range kernel element (BE) array module sequentially to produce the 2D pixel window. When two lines of the line memory are full and three *I*s of the third line are stored, the state controller starts the operation of the 2D BE array module in order to compute (6). Subsequently, the spatial kernel unit is enabled after one clock. The $K_{C}$ interpolation unit is activated before three clock cycles of the start of the 2D BE array module to provide $K_{C}^{i}$ on time. As a result, the final output of the B-NABF is generated per clock.

The data, such as $K_{C}s$, α, and the spatial kernel weight ($f_{s}$ in (1)), are sent by the host CPU. The host interface module stores them in registers, and the data are sent to each unit.

### 4.2. Binary Range Kernel Unit

The binary range kernel unit computes (6) in parallel for all pixels within the window. To scale the size of the pixel window easily, a unit module, called the binary range kernel element (BE), is designed for computing (6) in one pixel, and the BE is duplicated and it is connected to process the pixel window in parallel, as shown in Figure 3. A subtractor calculates $I\left(x_{i}\right)-I\left(x\right)$ in the BE, and the absolute value is computed using the sign of its most significant bit (MSB) and a two’s complement. Furthermore, a comparator is employed to compare $|I\left(x_{i}\right)-I\left(x\right)|$ with $K_{C}^{i}$, and a multiplexer is connected to select the input intensity or 0 as an output ($O_{xi}$) according to the compared result. The compared result, which is 1 or 0, is sent to the spatial kernel unit as a valid signal ($V_{xi}$) with $O_{xi}$.

The line memory is designed using first-in first-out (FIFO) memories connected serially and a shift register in the BE in order to provide the intensities of the neighboring pixels to each BE in parallel. The output ports of each FIFO memory are connected to the input port of the BEs in the first column in the 2D BE array module, and intensities are shifted to the right BE while using the shift register connected horizontally, as shown in Figure 3. The output of the shift register in BE21 is used as the center pixel of the window (I(x)) and it is connected with all of the BEs.

In addition, the output of the third FIFO memory ($I_{20}$) is sent to the $K_{C}$ interpolation unit in advance to generate the $K_{C}^{i}$ corresponding to I(x) and synchronize it with $|I\left(x_{i}\right)-I\left(x\right)|$.

### 4.3. $K_{C}$ Memory & $K_{C}$ Interpolation Unit

A memory with data width of eight bits and a depth of 256 is used for the $K_{C}$ memory, because the intensity and its difference are between 0 and 255. Four memories are used to store $K_{C}$s of four camera gains. Furthermore, a logic in the host interface module is added to update the $K_{C}$ memory by the host CPU at the initialization.

The $K_{C}$ interpolation unit accesses the $K_{C}$ memory using $I_{20}$, which is sent from the binary range kernel unit, as an address, and the four $K_{C}$s corresponding to each camera gain are obtained. Here, $K_{C}^{i↓}$ and $K_{C}^{i↑}$ are selected among the four $K_{C}$s by a multiplexer and its selection signal ($K_{C}\_Sel$), which is computed in advance while using a current camera gain in the host CPU. Subsequently, (7) is computed using two parallel multipliers and an adder. The precomputed $\frac{G_{i↑}-G_{i}}{G_{i↑}-G_{i↓}}$ and $\frac{G_{i}-G_{i↓}}{G_{i↑}-G_{i↓}}$ of (7) are sent from the host CPU. Finally, to generate $K_{C}^{i}$, the output of the adder is summed with its MSB of the fractional part for a round operation.

### 4.4. Spatial Kernel Unit

The 24 neighboring pixels in the window are classified into six groups according to the distance from the center pixel. The spatial kernel element (SKE) module adds up the four $O_{xi}$s of each same group, and the $f_{s}$ of the group is computed by multiplying the sum with the spatial kernel weight that corresponded to the group. Furthermore, to obtain the normalization term ($W_{p}$) of each group, the four $V_{xi}$ values, which denote the valid pixel after conducting the binary range kernel, are summed up. According to the sum of $V_{xi}$s, $W_{p}$ is selected among the multiples of $W_{s}$, which are generated using the shift logic and the adder, as shown in Figure 3. Then the $f_{s}$ values and $W_{p}$ values computed by the six parallel SKE modules are added up, respectively, by the adder tree. Finally, a divider divides the sum of $f_{s}$s by the sum of $W_{p}$s, in order to generate the final output ($O_{B-NABF}$).

## 5. Experimental Results

### 5.1. Image Quality by Denoising

**Figure 5:**
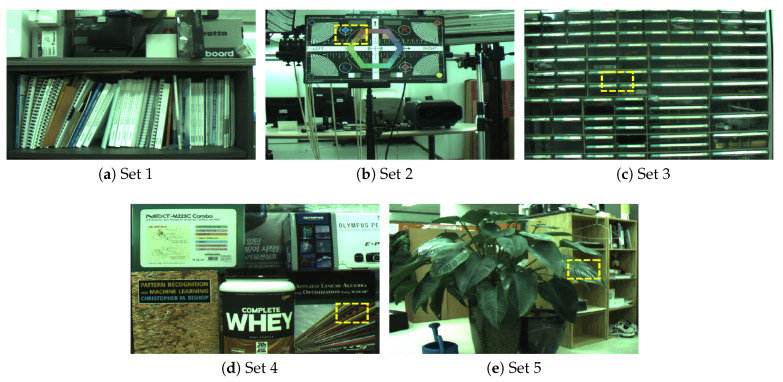
Dataset for the evaluation. As shown From (**a**) to (**e**), we captured highly-textured and various color-distributed scenes for evaluating the proposed method. Yellow-dashed rectangles are the regions to be enlarged for the qualitative comparison presented in Figure 6.

The quality of images are evaluated, which were filtered by the proposed NABF and B-NABF, via PC simulation before designing a dedicated VLSI. Five highly-textured test datasets are created, which were captured by a PointGrey Flea3 camera, as shown in Figure 5. Furthermore, a scene is captured 1,000 times and averaged the images in order to produce ground truth images. Six camera gain settings are used to verify the effect of noise variation. In addition, $\sigma_{s}$, $\sigma_{r}$, and α were varied to evaluate the quality variation according to the parameter setting.

Table 1 shows the average peak signal-to-noise ratios (PSNRs) of the conventional bilateral filter (BF), the proposed NABF, and B-NABF. The 15(I) of camera gain settings used the $K_{C}$s, which were interpolated using (7) based on measured $K_{C}$s in 10 and 18. The NABF improves the PSNR when compared with the input image under all noise conditions and parameter changes. However, the conventional BF decreases the PSNR when the noise level is low. Furthermore, the PSNR degradation by B-NABF and by using interpolated $K_{C}$s is negligible when compared with that by NABF and when measured $K_{C}$s were used, despite the approximation. In addition, the conventional BF is quite sensitive to variation of $\sigma_{s}$ and $\sigma_{r}$. Whereas, the PSNR by NABF is stable despite variations of $\sigma_{s}$ and α. For instance, the PSNR differences of the NABF and the conventional BF according to the parameter setting are 0.2 dB and 6.4 dB, respectively, when the camera gain is 0 dB.

Table 2 shows the averaged values of PSNR of all camera gains for each test set. With scene variation, the NABF and B-NABF are also more robust to parameter variation than the conventional BF, and the PSNR is improved.

**Figure 6:**
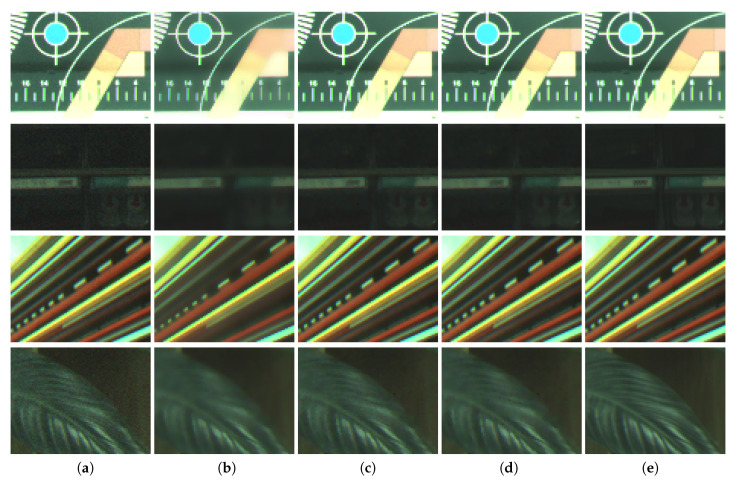
Qualitative comparison: (**a**) input images, the results of (**b**) the conventional bilateral filter, (**c**) the proposed NABF, (**d**) B-NABF, and (**e**) the ground truth

Figure 6 presents the qualitative results of the BF, the proposed NABF, and B-NABF with the ground truth images. The NABF shows clearer edges and textures similar to those of the ground truth images, while the conventional BF produces less distinct results. Furthermore, the results of the B-NABF are almost the same as them of the NABF.

### 5.2. Implementation Result and Comparison

Based on an architecture proposed in Section 4, a VLSI of B-NABF is designed while using Verilog hardware description language. It is verified that the register-transfer level (RTL) simulation results of the designed VLSI coincide with simulation results from the C model of B-NABF which is used in Section 5.1. Finally, the verified VLSI design was implemented in a Xilinx Virtex 7 FPGA (XC7VX330T).

#### 5.2.1. System Configuration for Measurement

**Figure 7:**
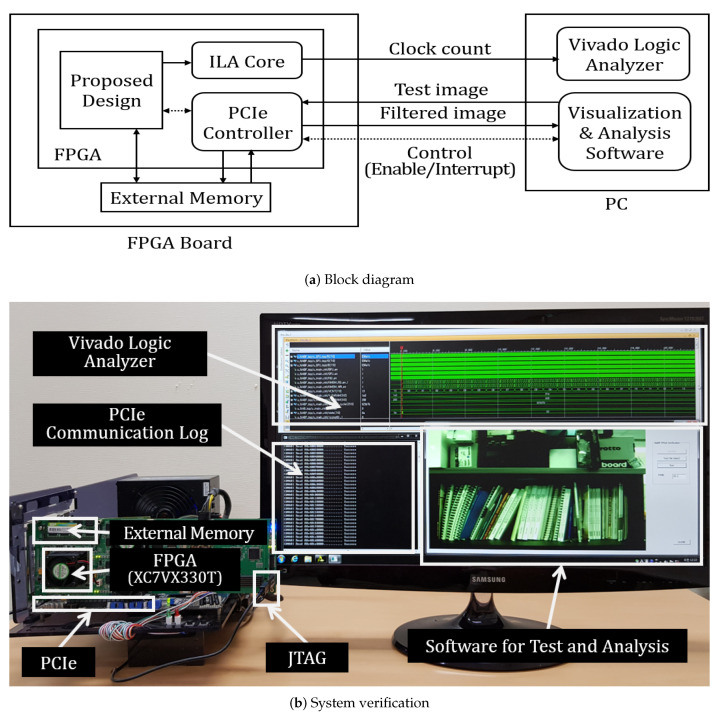
System configuration for the experiment.

A PC is connected with a FPGA board using a peripheral component interconnect express (PCIe) to verify the proposed VLSI design and measure its performance, as shown in Figure 7. A test image from the PC is stored in an external memory of a FPGA board, and the VLSI design is enabled by the register setting from the PC. After bilateral filtering is finished, an interrupt signal is generated by the main controller of VLSI and then sent to the PC. The interrupt service routine reads the filtered image from the external memory and displays it. The PSNR is calculated by comparing the filtered image with the ground truth image. To execute these processes automatically, a software programs for visualization and analysis is implemented, as shown in Figure 7b. Moreover, the throughput is measured using a clock counter, which is placed in the proposed design. The total number of clocks is probed using Xilinx integrated logic analyzer (ILA), and it is displayed in the Vivado logic analyzer via joint test action group (JTAG) interface. The logic and memory usage are reported after post-implementation in the Vivado design suite tool.

#### 5.2.2. Comparison with Recent VLSI Designs of Bilateral Filter

As in Table 3, VLSI designs [12,13] are considered that do not use an external memory in this study, because designs [10] that use an external memory are inappropriate for mobile systems due to the large power consumption. One earlier design [12] handles a large image in realtime owing to parallelized and pipelined implementation of (1). Pixels in a pixel window are grouped according to the distance from the center, and the pixels in each group are processed subsequently in a separate computing pipeline in order to reduce the usage of the logic and internal memory. However, the throughput is reduced due to the sharing of computing pipeline. Another design [13] implements an iterative algorithm of the bilateral filter, which was presented in [23], in order to handle an arbitrary pixel window size. However, the throughput is reduced despite a small image size due to the iterative nature of this algorithm. Moreover, a large amount of the internal memory is used for the storage of intermediate data between iterations.

In contrast, the throughput of the proposed VLSI design is 10.5 and 95.7 times higher than those of [12,13], respectively, owing to the proposed binary noise-aware bilateral filtering scheme. Furthermore, 63.6% and 97.5% less internal memory are used in comparison to [12,13], respectively. The logic usage is also significantly reduced. In terms of the image quality after filtering, as described in Section 5.1, the proposed noise-aware bilateral filter provides more stable and better quality under various noise conditions in comparison to the conventional bilateral filter adopted in [12,13].

#### 5.2.3. Integration of Implemented VLSI Design and Image Sensor

**Figure 8:**
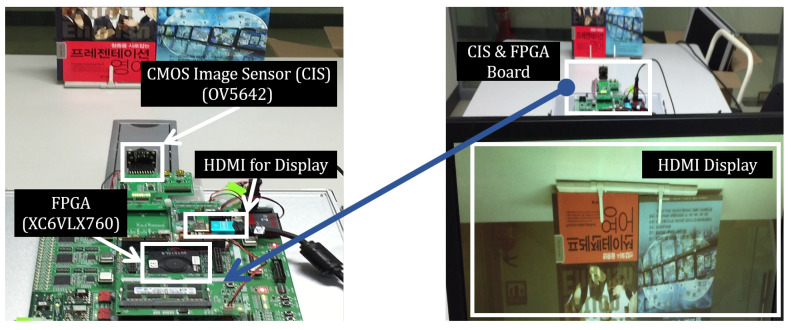
System configuration for integrating the implemented VLSI design of the proposed filter with CMOS image sensor.

A rapid prototyping system (http://huins.com/en/m11.php?m=rd&no=86) is used, which is based on Xilinx-6 XC6VLX760 FPGA, to integrate implemented VLSI design and CMOS image sensor (CIS). Omnivision OV5642 CIS is connected with the VLSI design in XC6VLX760 FPGA, and filtered output image is displayed in a monitor via high definition multimedia interface (HDMI), as shown in Figure 8.

**Figure 9:**
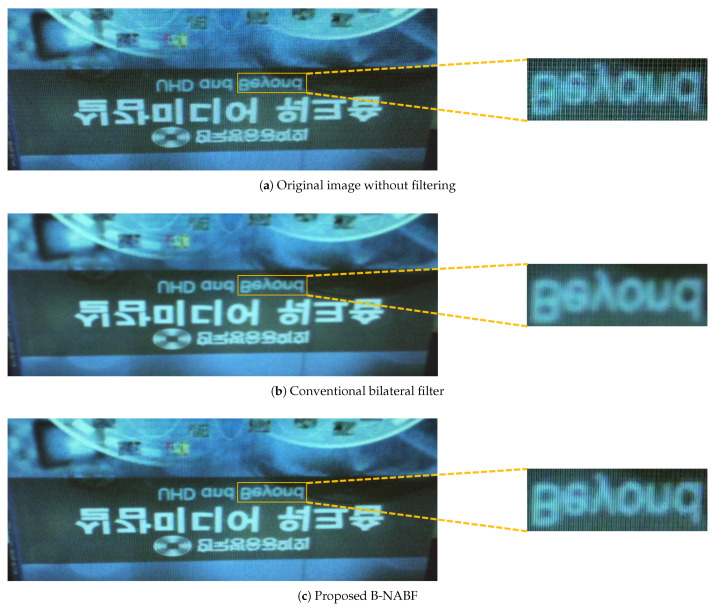
Filtered images by VLSI designs of the conventional bilateral filter and the proposed B-NABF.

In addition, a VLSI design of the conventional bilateral filter is implemented for comparing with a result of the proposed VLSI design qualitatively. As a result, an image that is filtered by the proposed VLSI design shows clearer edges and textures when compared with an image from a VLSI design of the conventional BF, as shown in Figure 9.

Figure 9 presents test results, which are filtered by the conventional bilateral filter and the proposed B-NABF, respectively, with the original image. A noise of the original image is reduced by a VLSI design of the proposed B-NABF. Furthermore, an image of the proposed VLSI design shows clearer edges and textures when compared with an image from a VLSI design of the conventional bilateral filter.

## 6. Conclusions

We have proposed a noise-aware bilateral filter (NABF) to overcome the disadvantage of the conventional bilateral filter in which image quality is degraded by randomly generated noise in image sensors. The NABF estimates the noise using an intensity difference-based image noise model and dynamically adjusts the weight of the range kernel according to the estimated noise. In addition, a light weighting scheme is introduced, which approximates the range kernel of the NABF into a binary kernel while using the statistical hypothesis test method, for resource-limited mobile systems. Finally, a fully parallelized and pipelined VLSI architecture of NABF based on the proposed binary range kernel is presented, which was successfully implemented in a FPGA. Our experimental results demonstrated that the proposed NABF is more robust to noise than the conventional bilateral filter under various noise conditions. Furthermore, the proposed VLSI design of the NABF achieves 10.5 and 95.7 times higher throughput and uses 63.6%–97.5% less internal memory than recent designs of the bilateral filter.

## Figures and Tables

**Table:** 


(**a**) Difference-based image noise model (**b**) Dependency on pixel intensity and camera setting

**Table:** 

**Table:** 

		
(**a**) Set 1	(**b**) Set 2	(**c**) Set 3

**Table:** 

	
(**d**) Set 4	(**e**) Set 5

**Table:** 





(**a**) (**b**) (**c**) (**d**) (**e**)

**Table 1:** Average peak signal-to-noise ratio (PSNR) under Various Noise Conditions.

Parameter		Input	Conventional BF			NABF		B-NABF	
$\sigma_{s}$		N/A ${}^{†}$	1.0	1.0	2.0	1.0	2.0	1.0	1.0
$\sigma_{r}$			0.04	0.08	0.08	N/A		N/A	
α			N/A			N/A		0.1	0.05
Gain [dB]	0	45.1	42.3	38.9	35.9	45.7	45.5	45.5	45.3
	5	43.7	42.0	38.2	35.4	44.4	44.2	44.2	44.1
	10	42.1	41.7	38.0	35.3	43.1	42.8	42.8	42.7
	15	39.3	40.0	37.4	35.0	40.5	40.2	40.2	40.1
	15(I) ${}^{‡}$	N/A				40.4	40.2	40.1	40.1
	18	36.7	37.7	36.0	34.1	37.8	37.5	37.6	37.5

^†^ N/A = Not Applicable, ${}^{‡}$ The 15(I) of camera gain settings used $K_{C}$ values, which were interpolated using (7) based on measured $K_{C}$s in 10 and 18.

**Table 2:** Average PSNR with Scene Variation.

Parameter		Input	Conventional BF			NABF		B-NABF	
$\sigma_{s}$		N/A ${}^{†}$	1.0	1.0	2.0	1.0	2.0	1.0	1.0
$\sigma_{r}$			0.04	0.08	0.08	N/A		N/A	
α			N/A			N/A		0.1	0.05
Test Set	#1	42.4	41.9	38.5	35.7	43.5	43.2	43.2	43.1
	#2	38.9	38.9	36.9	34.7	39.6	39.4	39.5	39.5
	#3	42.5	41.7	37.9	35.3	43.7	43.4	43.4	43.4
	#4	40.6	39.7	36.7	34.5	41.2	41.0	40.9	40.8
	#5	42.5	41.5	38.5	35.6	43.4	43.1	43.0	42.9

^†^ N/A = Not Applicable.

**Table 3:** Comparisons with State-of-the-Art VLSI Designs of Bilateral Filter.

		[12]	[13]	Proposed
Device		Xilinx-5 FPGA	Xilinx-7 FPGA	Xilinx-7 FPGA
		(XC5VLX50)	(XC7Z020)	(XC7VX330T)
Image Resolution		1024×1024	256×256	1920×1080
Max. Freq. (MHz)		320	63	330
Throughput (Mpixels/s)		31.5	3.45	330
Logic Usage (ea)	Slice ${}^{‡}$	1060	†	476 ${}^{a}$
	LUT	†	2647	1425
	FF	†	686	552
	DSP	29	10	8
Memory Usage (KByte)		49.5	706.5	18

^†^ It is not reported, ${}^{‡}$ It consists of multiple LUTs and FFs, ${}^{a}$ Synthesis result in XC7VLX50 (Xilinx-5 FPGA) for a fair comparison with [12].
